# Herb-Induced Liver Injury by Cimicifuga racemosa and Thuja occidentalis Herbal Medications for Fertility

**DOI:** 10.1155/2021/8858310

**Published:** 2021-01-18

**Authors:** Rohan Patel, Fareeha Alavi, Susel Ortega, Ajsza Matela

**Affiliations:** ^1^American University of the Caribbean, School of Medicine, 880 SW 145th Avenue, Suite 202, Pembroke Pines, FL 33027, USA; ^2^Department of Internal Medicine, BronxCare Health System, 1650 Grand Concourse, Bronx, NY 10457, USA; ^3^Division of Pulmonary and Critical Care Medicine, Department of Internal Medicine, BronxCare Health System, 1650 Grand Concourse, Bronx, NY 10457, USA

## Abstract

Herb-induced liver injury (HILI) is often an underreported sequela for many herbal remedies due to the lack of safety measurements involving these supplements. *Cimicifuga racemosa* and *Thuja occidentalis* are two herbal medications commonly used by women for fertility purposes. Many herbal preparations of these two supplements do not specify the risks behind their individual usage. We present a case of a 40-year-old woman who developed acute liver injury after concomitant use of these two products assessed for causality using the updated RUCAM. Upon a detailed investigation, the patient did not have evidence of underlying liver disease or any other risk factors to explain her presentation. After discontinuation of both herbal supplements, the patient had complete resolution of her symptoms and a significant improvement of transaminitis. This report highlights the importance of potential risk of hepatotoxicity induced by concomitant use of *Cimicifuga racemosa* and *Thuja occidentalis*.

## 1. Introduction

The use of herbal medications is increasing significantly around the world as they are frequently considered “natural” products. There is a perception that herbal preparations are without adverse effects [1]. Black cohosh (*Cimicifuga racemosa*) is one of the most commonly used herbal products, which is a perennial plant native to North America. It is primarily used for fertility and perimenopausal symptoms [2]. Arborvitae or white cedar (*Thuja occidentalis*) is a native European tree widely used in homeopathy and phytotherapy. It is used as a mother tincture or dilution. In combination with other herbs, white cedar may be used for acute and chronic infections of the upper respiratory tract and as an adjuvant to antibiotics. In folk medicine, it was utilized to treat uterine carcinomas and amenorrhea [3]. In this report, we present a case which highlights potential risks of herbal remedies, *Cimicifuga racemosa* and *Thuja occidentalis*, which were used in combination and caused acute liver toxicity.

## 2. Case Presentation

A 40-year-old woman with a past medical history of gastroesophageal reflux presented to emergency department with three days of severe abdominal pain localized in the right upper quadrant and epigastrium. The pain was continuous, nonradiating, and aggravated by food. She took omeprazole with no symptomatic relief. The patient also reported malaise and subjective fevers. She had no vomiting or diarrhea and denied sick contacts or eating undercooked or stale foods. She had no previous surgeries or blood transfusions. She is a homemaker, originally from Bangladesh where she traveled approximately one month prior to admission. She is a lifetime nonsmoker and denied alcohol or illicit drug use.

**Figure 1:**
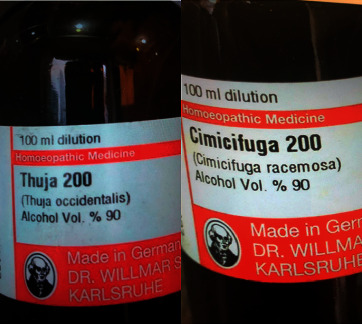
Patient's medication bottles.

During detail questioning, the patient admitted that she is trying to conceive a child with her husband. As per her friend's recommendation, she was taking herbal medications *Cimicifuga racemosa* and *Thuja occidentalis* for one month prior to onset of her symptoms. Patient's medication bottles are shown in Figure 1. We recommended her to stop all herbal supplements on admission.

On presentation, the patient was afebrile with a temperature of 98.6°F, blood pressure of 148/95 mmHg, pulse of 94 beats per minute, and oxygen saturation of 100% on room air. On physical examination, the patient was awake, alert, and comfortable. Heart and lung exam were unremarkable. Abdomen was soft and nontender, and bowel sounds were normal. She had anicteric skin and mucous membranes and no peripheral edema.

Initial laboratory findings were significant for transaminitis, as shown in Table 1. Acetaminophen level was less than 14.9 mcg/mL (normal values 0–30 mcg/mL), which indicates nontoxic levels of acetaminophen, correlating to the patient's lack of usage history. Ferritin and transferrin saturation were within normal limits. Lipase level was 105 (normal value is less than 61 U/L). Autoimmune and viral hepatitis workup was unremarkable, as shown in Table 2. Complete blood cell count was normal. Urine toxicology test was negative, and serum ethanol level was less than 10 mg/dL.

**Figure 2:**
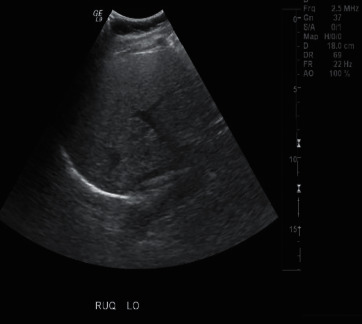
Abdominal ultrasound showing fatty liver.

The patient was started on intravenous fluids and intravenous ceftriaxone for suspected acute cholecystitis. Ultrasound of abdomen showed no evidence of cholelithiasis or cholecystitis and revealed fatty liver, as shown in Figure 2. Computed tomography scan of the abdomen showed no evidence of acute intra-abdominal findings. Gastroenterology team was consulted and recommended magnetic resonance cholangiopancreatography (MRCP) of the abdomen which showed no biliary pathology. Furthermore, the patient underwent hepatobiliary iminodiacetic acid (HIDA) scan which was negative for cholecystitis, and antibiotics were discontinued.

Patient's symptoms improved significantly within five days of the hospitalization and her liver tests (LTs); specifically, AST and ALT continued to normalize, as shown in Table 1. The patient's liver function tests (LFTs), which include total and conjugated bilirubin, INR, and GGT, did not indicate any functional abnormality in the liver. Liver biopsy was deferred, and the patient was discharged home in a stable condition. During follow-up appointment 2 weeks postdischarge, she had complete resolution of symptoms.

## 3. Discussion

Herbal supplements have continued to gain traction in the last ten years and are being used by approximately 20% of the population [4]. In a study by Rashrash et al., 35% out of the total 26,157 respondents had reported current usage of at least one herbal medication [5].

The Food and Drug Administration (FDA) considers “botanical” or herbal products dietary supplements [6]. Therefore, they are not subject to the same regulations, testing, and labeling as drugs. Despite their common use, there are insufficient data and evidence to demonstrate the safety and efficacy of most herbal products [4].

Historically, black cohosh (*Cimicifuga racemosa*) has been used for a variety of conditions including bronchitis, rheumatism, anxiety, fever, and snakebite. At present, it is most commonly used for perimenopausal symptoms [1, 7]. Several studies restated that black cohosh lacks estrogenic properties and appears to act as mixed competitive agonist of serotonin receptors (5HT1A and 5HT7) which are associated with hypothalamus and thermoregulation [2, 7].

Reported adverse effects range from most commonly mild reactions such as gastrointestinal symptoms, rashes, headaches, and dizziness to acute liver damage and even death [2, 8–10]. There have been reports of hepatotoxic effects from steatosis, liver necrosis, and fulminant hepatic failure requiring liver transplantation [1, 8, 10, 11]. The mechanism of acute liver injury is unclear. Black cohosh contains catechols and phenols, which can be activated to electrophilic quinone metabolites and trapped by glutathione or other sulfhydryl causing free radical mediated injury [9]. Studies have shown that mechanisms of liver injury involve toxic necrosis and are found often in autoimmune hepatitis or steatohepatitis [8, 12]. The discontinuation of black cohosh ingestion leads to rapid resolution of hepatic injury, indicating the importance of early clinical suspicion of the cause of liver damage [9].

Similarly, to black cohosh, toxicity from Arborvitae (*Thuja occidentalis*) can cause wide range of side effects. Some of them include gastrointestinal upset, headaches, agitation, convulsions, liver and kidney damage, myocardial injury, and arrhythmias [3]. *Thuja occidentalis* has been mostly related to neurotoxicity by affecting gating properties of GABA-A chloride channels. Animal models have also suggested that thujone may be porphyrinogenic [3]. One of the herb main extracts is alpha- and beta-thujone, that leads to proinflammatory effects on the body [13]. In vitro studies have shown increased cytokine induction, CD4+ T cell differentiation, and activation, antibody production, and NO_2_ production in macrophages [3]. Other studies notably reported that the chronic toxicity reaches the liver tissues [13].

A careful review of previously published case reports and systematic reviews of individual uses of *Cimicifuga racemosa* and *Thuja occidentalis* shows some temporal relationships between liver injury but lacks a data-driven method to place causality for herb-induced liver injury [1–3, 8, 10, 11, 13]. Rolf et al. review nearly 69 retrospective, prospective, and meta-analyses studies that claim some of the suspected correlations between usage of *Cimicifuga racemosa* and hepatotoxicity; however, many of the claims are unfound due to lack of proper protocols of causality. One case report by Adnan et al. did use a previously accepted scoring by the Council for the International Organizations of Sciences (CIOMS) and found a probable evidence of +9 with *Cimicifuga racemosa* and hepatotoxicity [9]. A systematic review by Naser et al. of 49-patient randomized, double-blinded, placebo-controlled trial found no reportable adverse effects with the use of *Thuja occidentalis*, while another study found 8 mild to moderate adverse events [3]. The review still supports some evidence of liver and renal toxicity with thujone, yet we continue to lack of evidence-based support for causality. Furthermore, with an extensive literature search on PubMed, no case reports have described concomitant usage of *Cimicifuga racemosa* and *Thuja occidentalis*, leading to an uncertainty of the drug-drug interactions that may be involved in our patient's presentation.

Rolf et al. assert the usage of the CIOMS scale for establishment of drug-/herb-induced liver injury. The CIOMS guidelines for liver injury first suggest excluding other etiologies of hepatic injury like viral hepatitides, alcohol-induced injury, hepatic and biliary outflow tract obstructions, ischemic necrosis, and autoimmune hepatitis [11, 14, 15]. The scoring can be done with the updated RUCAM (Roussel Uclaf Causality Assessment Method) system, which uses a scoring encompassing time to onset, course of the illness, risk factors, concomitant drug use, search for nondrug causes, previous drug-induced hepatotoxicity, and response to readministration of a drug or herbal supplement [14]. The standard initial criteria suggesting drug- or herb-induced liver injury include a presenting serum value of ALT > 5*N* or ALP > 2*N*, with *N* being the upper limit of the normal. Then, *R* value is calculated via *R* = ALT/ALP, which will help classify the injury based on a hepatocellular (*R* ≥ 5), cholestatic (*R* ≤ 2), or mixed liver injury pattern (2 < *R* < 5). Upon classification of the type of liver injury, the RUCAM system scale will score the likelihood of the causality. The scale ranges from −9 to +15 with the interpretation as follows: <0 is excluded, 1-2 is unlikely, 3–5 is possible, 6–8 is probable, and above 9 is highly probable [14, 15].

Based on the updated RUCAM system scale for causality assessment of drug-induced liver injury (DILI) and herb-induced liver injury (HILI) [14], our patient's calculated *R* of 2.33 suggested a mixed liver injury pattern and provided a RUCAM scaled score of +6. Therefore, we believe that the disease has probable cause by usage of *Cimicifuga racemosa* and *Thuja occidentalis*.

Our patient presented to emergency department with upper abdominal pain after using Cimicifuga 200 and Thuja 200 for one month. Detailed history confirmed no concomitant ingestion of other hepatotoxic substances. Multiple laboratory tests and imaging studies excluded other potential causes of acute liver injury. The patient showed remarkable clinical improvement and normalization of liver function tests after discontinuation of herbal supplements.

Danan and Teschke indicate that a diagnosis of herb-induced liver injury necessitates the challenge, dechallenge, and rechallenge of the herb with subsequent repeated measurements of liver function tests [14]. Our patient's liver function significantly improved after discontinuation of both herbal supplements. We decided against rechallenging and recommended that she permanently stops both herbal supplements. As *Cimicifuga racemosa* and *Thuja occidentalis* extracts were self-administered by the patient with an intention to conceive a child, she was referred to fertility clinic for further treatment.

In this case, if rechallenging was to be performed, it would require separation of both herbal supplements first with subsequent administration of both of them together. It is unclear if patient's liver dysfunction was caused by one of the herbal substances or a combination of both administered concomitantly.

To the best of our knowledge, this is the first reported case of concomitant use of *Cimicifuga racemosa* and *Thuja occidentalis* for fertility treatment causing acute liver toxicity.

## 4. Conclusion

Our case report emphasized the importance of detail history-taking that includes inquiring about use of herbal supplements, as these “natural” remedies may be associated with significant risks. Most cases of herb-induced liver injury (HILI) are benign and improve after drug withdrawal. Early recognition and discontinuation of the offending agent is essential to prevent progression to life-threatening liver disease. Standardized labeling of herbal medications with information about side effects and warning against concomitant use with other medications and herbal supplements could potentially prevent serious adverse reactions.

## Figures and Tables

**Table 1:** Liver function tests.

Laboratory tests	Normal values	Values on admission	Values at discharge
Alanine transaminase (ALT)	5 to 40 U/L	238 U/L	145 U/L
Aspartate transaminase (AST)	5 to 40 U/L	203 U/L	129 U/L
Alkaline phosphatase (ALP)	35 to 130 U/L	102 U/L	95 U/L
Total bilirubin	0.1 to 1.2 mg/dL	0.6 mg/dL	0.4 mg/dL
Gamma-glutamyl transferase (GGT)	10 to 48 U/L	124 U/L	—
INR	<1.10	1.06	1.10

U/L = units per liter; g/dL = grams per deciliter; mg/dL = milligrams per deciliter.

**Table 2:** Autoimmune and hepatitis panel.

Laboratory test	Immune status
Antimitochondrial antibodies (AMA)	Negative
Antinuclear antibodies (ANA)	Negative
p-ANCA	Negative
Anti-DNA antibodies	Negative
Anti-smooth muscle antibodies	Negative
Anti-liver kidney microsome type 1 antibodies	Negative
Anti-HAV IgM	Negative
HBsAg	Negative
Anti-HBs	**Positive**
Anti-HBc	Negative
Anti-HCV antibodies	Negative
Anti-HEV IgM	Negative
Anti-HEV IgG	Negative

## Data Availability

The data used to support the findings of this study are available from the corresponding author on reasonable request.
